# Quantifying Radiation Exposure in Minimally Invasive Spinal Surgery: A Single-Surgeon Study of Minimally Invasive Surgery-Oblique Lateral Lumbar Interbody Fusion (MIS-OLLIF) With Double C-Arm Technique

**DOI:** 10.7759/cureus.71933

**Published:** 2024-10-20

**Authors:** Hamid Abbasi, Dominic Moore, Jiawen Zhan, Twanesha Lightbourn, Adam Sima, Mitch A Rusten

**Affiliations:** 1 Spine Surgery, Inspired Spine Health, Burnsville, USA; 2 Machine Learning, Inspired Spine Health, Burnsville, USA; 3 Clinical Medicine, Inspired Spine Health, Burnsville, USA; 4 Clinical Medicine, Nura Pain Clinic, Burnsville, USA; 5 Research, Inspired Spine Health, Burnsville, USA

**Keywords:** lumbar fusion, minimally invasive lumbar approach, minimally invasive spinal surgery, occupational radiation dose, spine surgery

## Abstract

In the advancement of spinal surgery, minimally invasive techniques such as the oblique lateral lumbar interbody fusion (OLLIF) have emerged, offering improved clinical outcomes and the use of dual C-Arm intraoperative imaging. Despite the benefits, the radiation exposure to surgeons and staff from this dual-source imaging has not been thoroughly examined. This study aims to quantify the radiation exposure received by surgeons during OLLIF procedures, filling a gap in current research that primarily focuses on single-source imaging. Over 12 months, radiation doses were measured across 121 surgeries at four locations.

Data analysis included average radiation exposure per surgery and variations across surgical sites. The findings showed an average radiation emission of 198.78 mGy per OLLIF surgery, with the surgeon receiving approximately 0.06 mSv per surgery. Cumulative doses for the surgeon were below the safety thresholds set by European and American standards.

The significance of these findings lies in their contribution to the understanding of occupational safety in spinal surgery. The results indicate that with proper protective measures, such as lead aprons, the radiation exposure from the OLLIF technique is manageable within occupational limits. This study highlights the importance of monitoring radiation exposure and may influence the development of new guidelines and protective strategies in the field. Future research could expand the cohort of surgeons to validate these findings, develop new protective strategies, and explore radiation exposure in more complex multi-level fusions.

## Introduction

In the realm of spinal surgery, the advancement towards minimally invasive techniques has culminated in the development of innovative procedures such as the minimally invasive oblique trans-Kambin lateral-posterior lumbar interbody fusion (OLLIF). In addition to its improved speed and clinical outcomes, this technique stands out due to its unique intraoperative imaging setup, entailing the simultaneous use of two C-arms to provide both anteroposterior (A/P) and lateral views in real-time. This dual-planar visualization approach is crucial for the precise placement of surgical hardware, proper instrument orientation, and accurate identification of anatomical landmarks. This is far from uncommon in the field of spinal surgery, as intraoperative imaging is crucial to assuring accurate and safe placement of devices such as pedicle screws and intervertebral cages, as these instruments are delivered close to vital vasculature and nerves [1]. This form of imaging is typically done with a single device, such as a C-arm. The OLLIF’s imaging strategy uses two imaging sources and has parallels in orthopedic surgeries, such as calcaneal fixations and percutaneous approaches to proximal humeral fractures, yet its implementation in spinal surgeries is considered pioneering [2,3]. 

Because of the novelty of this approach, a critical element that has yet to receive attention is the radiation exposure experienced by surgeons and clinical staff during the OLLIF procedure, specifically from the use of two radiation sources [4]. Existing research predominantly focuses on radiation doses from single-source, single-plane intraoperative imaging, leaving a knowledge gap when it comes to dual-source biplanar exposure. Recognizing this deficit, the present study is designed to quantify the radiation exposure received by surgeons while performing the OLLIF technique. By employing two distinct dosimeters, we collected data on radiation doses across 121 surgical sessions and four locations over a period of approximately 12 months.

Given that the minimally invasive nature of the OLLIF procedure allows for a higher number of operations within a given timeframe when compared to traditional methods, it is imperative to ascertain the implications of this increased surgical volume on radiation safety for healthcare professionals [5]. Other inquiries into radiation doses show exposures for spine surgeons ranging from 0.16 mSv (millisievert) to 2.29 mSv with single C-arm approaches [6]. A similar study was conducted with orthopedic surgeons and a single C-arm, showing that they too fell well within permissible limits [7]. Fields that use intraoperative more frequently, such as interventional cardiology, show annual exposure averages estimated at around 5 mSv [8].

The anticipated outcomes of this research are vital; they hold the potential to significantly enhance our comprehension of the occupational hazards, such as cancer or cataracts, linked with advanced surgical practice and to develop strategies to mitigate these risks [9]. In doing so, we aim to contribute invaluable insights into the equilibrium between surgical innovation and personnel safety in modern spinal surgery.

## Materials and methods

An observational study was conducted to collect images using dual C-Arm fluoroscopy from a total of 121 OLLIF procedures over a period of approximately 12 months. All procedures took place across four different surgical sites (Alomere Health, Inspired Spine Surgical Center, Riverview Healthcare, and Summit Surgical) and were performed by a single surgeon to minimize variance in participant location during surgery and frequency of exposure. Inclusion criteria were any OLLIFs performed during the 12-month time period, while any other case types were excluded. The images acquired included lateral views, as well as A/P views, which facilitated the examination of the procedural technique and anatomy from different angles. Final images were also obtained before closing to be certain of positioning. During these periods of imaging, the surgeon, and therefore dosimeters, were within 6 ft of exposure to the imaging C-arm. The C-arms used across the sites of this study were all GE OEC 9800 or 9900 models (GE, Salt Lake City, UT), which only differ in screen display interface, not imaging quality or the levels of radiation emitted. 

During the OLLIF procedures, radiation exposure was quantitatively measured for the surgeon using two Luxel®+ dosimeters (Landauer, Glenwood, IL) or two optically stimulated luminescence (Radiation Detection Company, Georgetown, TX) placed on specific regions of the body: one on the left chest and another on the hip area. These sensors were strategically placed to monitor the exposure levels accurately and were analyzed every three months to ensure consistent data recording between the two dosimeters. During these procedures, the operator also wears a standard radiation-resistant lead apron with thickness between 0.35 mm and 0.5 mm radiation, as traditional modalities of stepping behind a lead shield or sequestering in a control room during imaging are not as viable during this surgery [10,11].

The reading of the dosimeters was independently analyzed at a centralized location that was separate from all surgical facilities involved in the study. The data retrieved from the dosimeters was then pooled together for a comprehensive general analysis. This assessment aimed at determining the average radiation exposure per OLLIF surgery and examining the variations in radiation exposure across the different surgical sites. 

Throughout the study period, meticulous efforts were made to standardize all processes and maintain quality control. Across the various surgical sites, the same methodology for image collection and radiation exposure measurement was employed. This standardization was critical in assuring the reliability of data and reducing the potential bias that could arise from procedural differences. Due to the absence of a used patient population and this being a single surgeon participant who was involved with the design of this study, no informed consent or additional ethical review was required. All data we obtained without any impact or change to procedure and patient’s care whatsoever and thus an IRB exemption was obtained. In addition to standard mean and deviation calculations, ANOVA (analysis of variance) was performed to evaluate for p-values and statistical significance.

## Results

**Figure 1:**
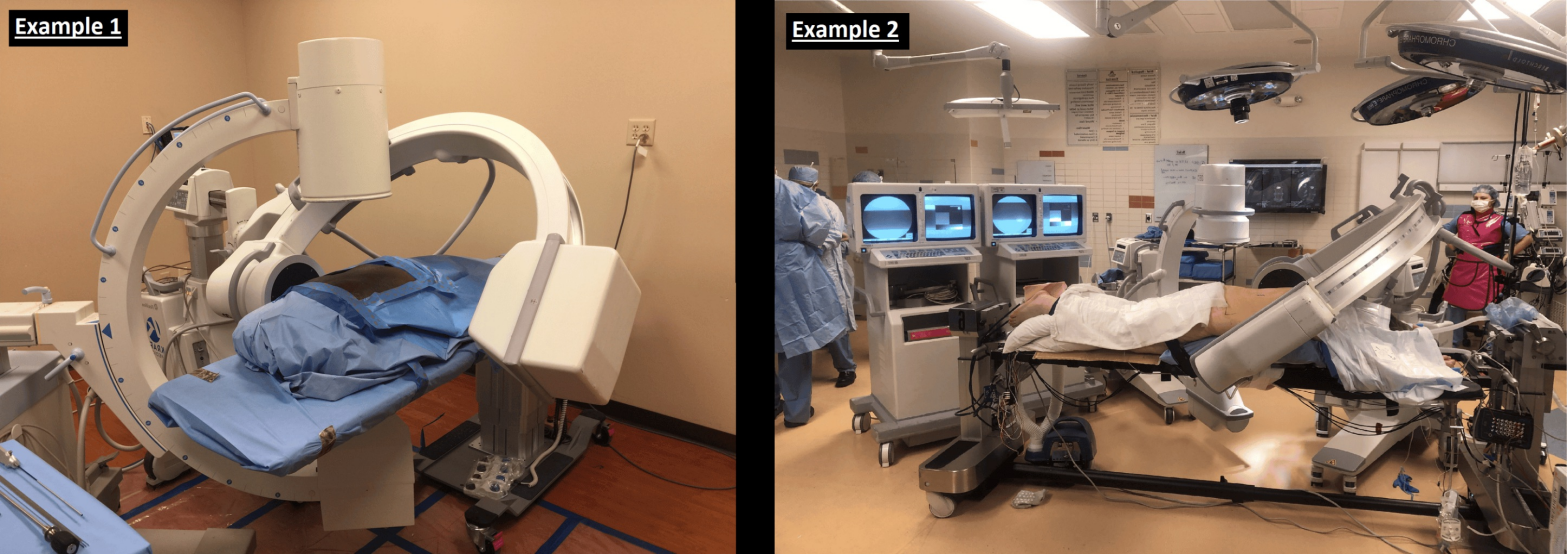
Intraoperative imaging setup used during the OLLIF procedure. One C-arm is placed laterally and angled approximately 45% towards the head of the bed, while the remaining is positioned across from the surgeon for an A/P view. OLLIF, oblique lateral lumbar interbody fusion; A/P, anteroposterior

**Figure 2:**
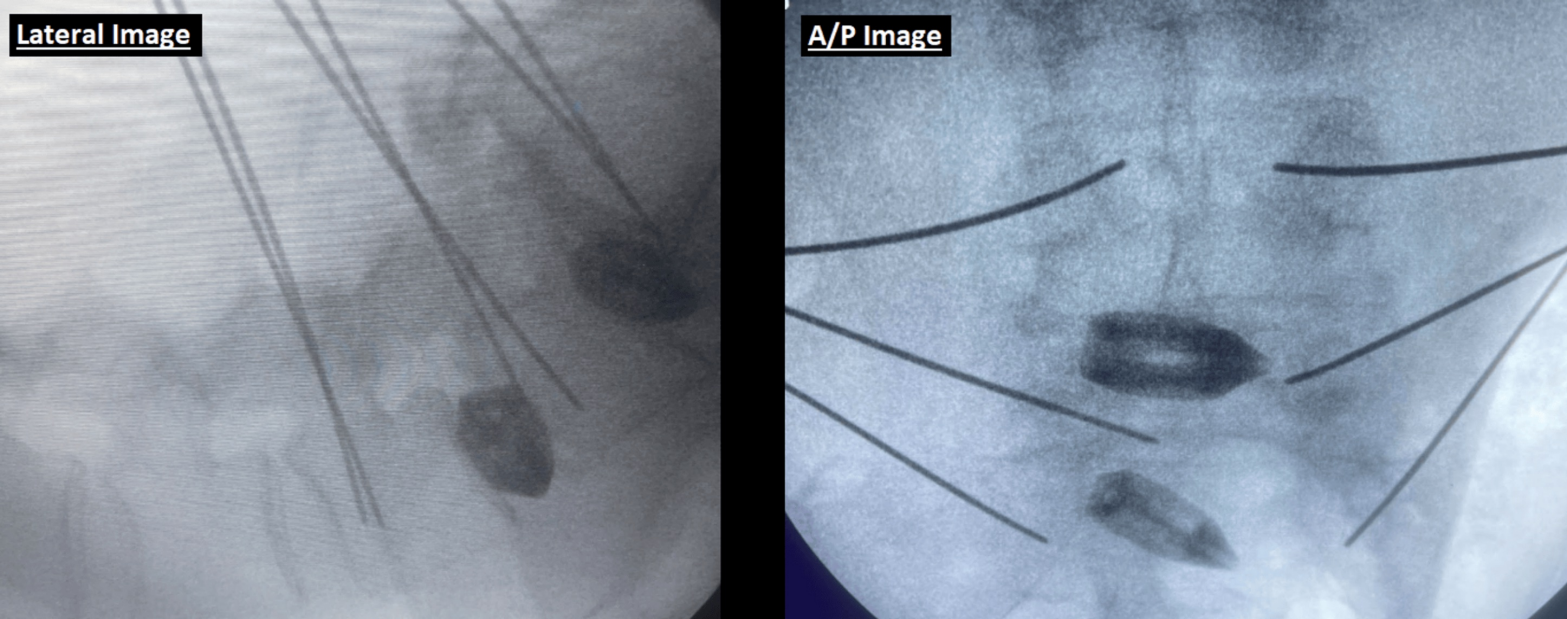
Views obtained from both the lateral and A/P C-arms used to gauge appropriate instrument placement. A/P, anteroposterior

During the operative time period under review, a total of 121 OLLIF surgeries were performed across four surgical sites and then reported to a central location. The data collected from these procedures included fluoroscopy times for both lateral and A/P views, the setup for which are depicted in Figures 1, 2, as well as the corresponding radiation doses measured in milligray (mGy). Tables of collective raw data can be found in the Appendix A and Appendix B. Of note, there are not enough data points for comparison of significance between chest and waist dosimeters, and thus they have been omitted at this time.

Table 1 shows a direct comparison of data between the four facilities used. This, coupled with the following data analysis, shows statistical significance between the facilities for total radiation emissions (p-=0.0298). There is no statistical significance between the facilities for the rate of radiation emission per minute of operative time (p=0.6443) and the rate of radiation per second of fluoroscopy time (p=0.3112).

**Table 1:** Average total doses of radiation from the C-arms at each facility, as well as the radiation emission per minute of operative time and per second of fluoroscopy time.

Facility	Average Total Radiation Emitted	Radiation Emission Per Minute of Operative Time	Radiation Per Second of Fluoroscopy Time
Facility 1	256.08	3.90	0.65
Facility 2	180.81	4.02	0.98
Facility 3	217.57	3.98	0.71
Facility 4	137.79	2.88	0.95

Table 2 presents the cumulative results of the fluoroscopy times and radiation doses. The average fluoroscopy time for lateral imaging was found to be 2.8 minutes, with a median of 2.44 minutes. For A/P imaging, which is used only at the beginning of the procedure during K-wire placement, the average time was 1.85 minutes, with a median of 1.66 minutes. These imaging durations were within the context of average operating times of 60 minutes and median operating times of 54 minutes. The total radiation dose emitted averaged at 198.78 mGy, with a median dose of 145.25 mGy across all surgeries.

**Table 2:** Cumulative C-arm readings for both lateral and A/P times and doses, with extracted averages, medians, and standard deviations. In addition, operative time is included here for ease of comparison. A/P, anteroposterior

Operative Data	Lateral Time (min)	A/P Time (min)	Operative Time (min)	Lateral Dose (mGy)	A/P Dose (mGy)	Total Dose (mGy)
Cumulative	503.47	332.24	10,620	15697.62	8355.88	24053.5
Average	2.80	1.85	60	129.73	69.06	198.78
Median	2.44	1.66	54	98.6	55.79	145.25
Standard Deviation	1.43	0.96	24.33	109.04	54.67	145.71

For the operating surgeon, the dosimeter readings indicated a deep dose or whole body equivalent of 7.43 mSv, a lens dose equivalent of 7.76 mSv, and a shallow dose equivalent of 7.52 mSv. Again, these readings were taken over the course of the 121 OLLIF procedures, and no additional surgeries performed during the time period in review were recorded with these dosimeters. On average, this resulted in approximately 0.06 mSv of received radiation per surgery for whole body exposure. These findings on radiation exposure are detailed in Table 3.

**Table 3:** Cumulative and average dosimeter readings over the 12-month period. In this table, the cumulative and average dosimeter readings over the 12-month period are shown for deep tissue, eyes, and the skin, and then compared to the total number of surgeries (121) and the total fluoroscopy time (31,102 seconds) to find the averages. DDE, deep dose equivalent; LDE, lens dose equivalent; SDE, shallow dose equivalent

Collective Dosimeter Readings	DDE mrem/mSv	LDE mrem/mSv	SDE mrem/mSv
Cumulative	743/7.43	776/7.76	752/7.52
Average per surgery	6.14/0.06	6.41/0.06	6.21/0.06
Average per second of fluoroscopy time	0.02389 mrem	0.02495 mrem	0.02418 mrem

This was further extrapolated to give estimates for received radiation based on the number of levels being addressed during each OLLIF. This is detailed in Table 4, which shows that a surgeon can roughly expect 0.04 mSv per one-level fusion, 0.06 mSv per two-level fusion, and 0.09 mSv per three-level fusion. Not enough data were available to posit on four-level and five-level fusions.

**Table 4:** Approximate averages of fluoroscopy time per level. Using these averages, a predicted average amount of exposure per surgery is calculated for each exposure type (DDE, LDE, and SDE) for the respective number of levels. DDE, deep dose equivalent; LDE, lens dose equivalent; SDE, shallow dose equivalent

Calculations per Level	One Level	Two Levels	Three Levels
Average fluoroscopy time	175.583	251.984	358.5
DDE mrem/mSv	4.19/0.04	6.02/0.06	8.56/0.09
LDE mrem/mSv	4.38/0.04	6.29/0.06	8.94/0.09
SDE mrem/mSv	4.25/0.04	6.09/0.06	8.67/0.09

## Discussion

The impetus for this study was to evaluate the cumulative radiation exposure experienced by a surgeon performing multiple OLLIFs across various sites within a single year and to determine the safety and sustainability of such exposure levels. Central to this inquiry is the need to assess the following: the issue of occupational radiation safety for surgeons, the potential solutions to mitigate any associated risks, and the novel insights our study provides. The statistical differences shown between facilities in terms of average radiation emission may be best attributed to differences in the complexities of surgeries with differing patient populations or additional factors such as operating room staff. Regardless, the lack of statistically significant differences in radiation emission per operative time and fluoroscopy time allows for all data to be used and analyzed collectively.

Our research offers a new perspective on the radiation doses received by surgeons performing OLLIF cases, showing that over the course of 121 surgeries, one can expect an average emission of 198.78 mGy, receiving an average dose of 0.06 mSv per surgery. The wide-ranging standard deviations seen with doses and times can be attributed to the varying complexities of different OLLIF cases with differing patient anatomies and the number of levels operated on. The radiation exposure expected per number of levels operated on is further analyzed to show that the more common one- and two-level surgeries deliver average doses of 0.04 mSv and 0.06 mSv respectively, while three-level cases deliver doses closer to 0.09 mSv. Surgeons who routinely operate on fewer levels can therefore expect to receive even less radiation than the surgeon in this study. Though there are not enough data to continue this line of investigation in four- and five-level OLLIF surgeries, one might reasonably expect the average radiation exposure to continue the increasing trend or else plateau at 0.09 mSv.

The noted cumulative doses range from 7.43 mSv to 7.76 mSv, which is consistently below the thresholds considered safe by both the European and American standards for whole body radiation, which are 50 mSv and 20 mSv, respectively [12,13]. Following the seen trend of mSv received per surgery, a conservative estimate would show that one could perform over 300 OLLIFs and still meet the European OSHA radiation standards. Previous studies have shown that in 2015, U.S. surgeons would average approximately 133.3 surgeries annually, while in 2017, Japanese surgeons would average 102.8 [14,15]. This includes a variety of procedures and not just lumbar fusions. It is worth noting that the radiation levels received by the surgeon in this study are above the dosage of radiation deemed safe for pregnant individuals annually [16]. 

The comparison of radiation levels in this study and those experienced by surgeons in interventional radiology is noteworthy. One study shows that these providers are subjected to mean annual effective doses ranging from 0.37 to 10.1 mSv [17]. The doses received by the surgeon in this study fall well within that range. Another aspect to consider is the comparison with other subspecialties such as vascular surgery, where average annual exposures, including complex and high radiation exposing procedures such as endovascular aortic repair (EVAR), account for only around 1% of the recommended limit [18]. Moreover, the protective measures employed during these surgical procedures, including the use of radiation shielding lead aprons, further reinforce the safety protocols in place to safeguard against excessive radiation exposure.

These comparisons underscore the relative safety of the radiation levels encountered in the studied surgical settings of the OLLIF. However, it is crucial to acknowledge that this study's scope is limited to a single surgeon's experiences, which may not capture the full spectrum of variability in surgical practices or the inclusion of other surgeries performed annually, though a previous study demonstrated that a single surgeon can expect to rapidly decrease their mean surgery time after performing even 10 consecutive one-level OLLIFs [4]. Outside of the variable of experience, the amount of imaging used, the duration of each procedure, and the preferred angles and approaches could differ significantly among surgeons, suggesting a need for broader studies to validate our findings. This incongruency was previously observed among surgeons evaluating radiation exposure during pinning of humeral fractures, showing that exposures to direct fluoroscopy varied from 0% to 97% depending on the surgeon and their positioning of themselves and the instrument [19] It is possible that the OLLIF procedure could experience similar variability, and thus it would be beneficial to expand the operating pool. 

At present, our study shows that current OLLIF surgical practices are effective in managing radiation exposure. It demonstrates that even with multiple surgeries and yearly accumulations, the radiation doses remain within recommended occupational limits. That said, the use of protective gear, such as lead shielding, plays a pivotal role in this safety profile, and all these devices should be regularly inspected and maintained. Future investigations could provide a more comprehensive understanding of radiation exposure across the surgical field with additional surgeons and potentially lead to innovations in protective measures or procedural techniques to reduce radiation exposure even further, as any amount is an increased risk for the operator and their cohort. The implications for clinical practice are clear: with appropriate safeguards, the levels of radiation exposure for surgeons, and therefore all surgical staff, can be managed effectively, ensuring the long-term occupational safety of these essential healthcare professionals, barring any stochastic events.

## Conclusions

This study aimed to evaluate the cumulative radiation exposure experienced by a surgeon performing multiple OLLIF procedures with protective equipment to determine the safety and sustainability of such exposure levels. Though there is always room for improvement, these findings suggest that the radiation exposure associated with the OLLIF procedure, even when performed frequently, can be safely managed within the recommended occupational limits set by national standards.

Future studies involving a larger cohort of surgeons are needed to validate the findings, include assessment of more four- and five-level fusions, and track trends in fluoroscopy time and operative time over a surgeon’s career to see if these decline and, if so, how rapidly. These findings underscore the importance of monitoring and managing radiation exposure in the operating room. They reassure that with appropriate protective measures, such as the use of lead aprons, the OLLIF technique can be performed safely with respect to radiation exposure to the surgeon and operating room staff. This contributes to the broader understanding of occupational safety in innovative surgical practices and may influence the development of new guidelines and protective strategies in the field of spinal surgery.

## Appendices

Appendix A 

**Table 5:** Dosimeter readings for all four sets of badges used during the time span of the study. DDE, deep dose equivalent; LDE, lens dose equivalent; SDE, shallow dose equivalent

Dosimeter Readings (in mrem)	DDE (1.0 cm)	LDE ("eye," 0.3 cm)	SDE (0.007 cm)
Facility 1	Beginning date 6/30/23 and ending date 6/30/24 (note, last surgery with this badge at facility 1 performed on 1/26/24)		
Lifetime as of 6/30/23	2,859	2,896	2,884
Lifetime as of 12/31/23	3,215	3,251	3,221
Collar	14	14	11
Waist	3	3	1
Lifetime as of 6/30/24	3,229	3,265	3,232
Facility 2	Beginning at badge inception date 4/10/23 and ending date 4/2/24		
Chest	76	71	58
Waist	22	21	20
Body (chest+waist)	98	92	78
Chest	89	85	75
Waist	24	23	19
Body (chest+waist)	113	108	94
Lifetime as of 4/2/24	187	177	153
Facility 3	Beginning at badge inception date 11/15/23 and ending date 3/14/24		
Control	15	15	15
Chest	29	29	27
Waist	1	1	1
Chest	26	26	24
Waist	3	3	3
Control	25	25	25
Chest	84	128	161
Waist	20	20	20
Lifetime as of 3/14/24	139	183	212
Facility 4	Beginning date 07/01/2023 and ending date 06/30/2024 (last surgery performed on 4/26/24)		
Chest	30	30	27
Chest	14	14	11
Waist	3	3	1

Appendix B 

**Table 6:** Data recordings of doses, operating time, fluoroscopy time, and the number of levels operated on during each case.

All Units Recorded in mGy	Lateral Dose	AP Dose	Total Dose	OP Time	Fluoroscopy Time	Number of Levels
Facility 1	182.1	73.09	255.19	65	409	2
	92.7	120.48	213.18	62	396	2
	334.76	218.11	552.87	86	524	3
	88.98	47.82	136.8	100	665	4
	102.75	60.77	163.52	68	368	2
	125.1	60.54	185.64	35	204	1
	176.91	175.06	351.97	50	357	1
	174.04	97.51	271.55	43	333	1
	75.94	36	111.94	66	415	2
	129.02	113.05	242.07	69	386	2
	142.76	37.32	180.08	59	341	2
	294.55	93.93	388.48	79	476	1
	47.23	22	69.23	93	209	2
	262.52	176.73	439.25	114	452	3
	51.27	55.24	106.51	45	244	2
	117.56	87.11	204.67	53	333	2
	169.1	181.75	350.85	100	529	5
	375.71	131.22	506.93	79	553	4
	108.43	90.88	199.31	66	424	3
	106.14	21.38	127.52	51	362	2
	78.89	59.08	137.97	27	185	1
	424.54	97.06	521.6	55	363	2
	77.81	51.37	129.18	128	333	2
	217.28	150.37	367.65	65	393	3
	297.9	376.3	674.2	82	585	3
	83.25	62	145.25	59	393	2
	180.1	17.19	197.29	83	511	3
	144.85	78.39	223.24	92	587	2
	41.4	27.61	69.01	117	176	3
	19.78	139.66	159.44	41	224	1
Facility 2	86.87	43	129.87	49	192	2
	46.59	23.85	70.44	95	268	2
	122.42	89.97	212.39	40	178	2
	38.27	21.83	60.1	30	100	1
	53.46	28.33	81.79	85	95	2
	68.2	74.46	142.66	32	101	1
	58.2	19.36	77.56	25	127	1
	76.5	29.36	105.86	45	323	2
	453.3	66.07	519.37	71	186	3
	37.5	17.51	55.01	67	364	3
	171.5	128.32	299.82	58	314	3
	209.1	116.32	325.42	87	532	3
	110.7	51.44	162.14	43	189	2
	650.1	277.8	927.9	29	112	1
	71.5	45	116.5	99	263	4
	82.7	52.38	135.08	39	296	1
	69.9	52.84	122.74	42	202	2
	349.9	47.9	397.8	49	190	2
	48.1	21.14	69.24	42	212	2
	192.9	32.43	225.33	35	176	1
	329.8	107.16	436.96	65	183	2
	45.26	69.5	114.76	43	288	2
	182.3	87.01	269.31	54	310	2
	13.01	21.9	34.91	44	168	2
	41.91	84.4	126.31	30	151	2
	28.88	95.3	124.18	44	182	2
	48.03	89.3	137.33	45	197	2
	69.4	90.9	160.3	52	186	2
	58.3	132.2	190.5	140	248	2
	31.6	15.1	46.7	39	185	2
	348.4	60.45	408.85	47	208	2
	23.97	72	95.97	26	152	1
	25.3	36	61.3	42	270	2
	63.78	76.2	139.98	66	347	3
	202.8	77.78	280.58	30	102	1
	21.75	59.2	80.95	29	103	1
	99.7	43.04	142.74	42	178	2
	50.15	176.5	226.65	86	185	3
	336.49	110.7	447.19	91	427	2
	189.86	43.98	233.84	47	218	2
	124.7	59.65	184.35	66	192	2
	103.6	26.3	129.9	49	202	2
	74.4	43.62	118.02	42	208	2
	103.7	117	220.7	43	223	2
	112.8	72.11	184.91	41	196	2
	98.7	53.7	152.4	45	209	2
	39.5	26.6	66.1	56	266	2
	34.6	38.96	73.56	50	175	3
	60.5	27.43	87.93	51	174	2
	45.9	27.31	73.21	40	152	2
	108.5	68.3	176.8	43	189	2
	65.2	33.98	99.18	48	197	2
	98.7	38.23	136.93	26	109	1
	101.6	34.91	136.51	40	159	1
Facility 3	95.54	26.61	122.15	103	179	5
	160.86	89.54	250.4	84	371	3
	64.57	18.74	83.31	50	219	2
	148.27	81.26	229.53	59	310	3
	171.87	76.85	248.72	45	263	2
	112.05	40.01	152.06	60	286	3
	132.45	77.55	210	41	279	2
	171.87	70.38	242.25	45	257	2
	259.1	172.45	431.55	79	551	3
	101.8	33.66	135.46	35	166	1
	200.19	91.55	291.74	64	355	3
	42.84	33.77	76.61	54	252	2
	93.76	127.33	221.09	35	306	2
	42.84	33.77	76.61	42	233	2
	72.43	26.35	98.78	26	143	1
	179.42	55.79	235.21	44	294	2
	151.66	86.32	237.98	76	506	4
	448.69	124.12	572.81	76	436	2
Facility 4	55.51	23.7	79.21	84	123	2
	105.99	28.8	134.79	60	201	2
	59.37	18.1	77.47	57	204	2
	120.26	64.1	184.36	44	130	2
	75.62	34.3	109.92	91	129	3
	74.65	36.2	110.85	49	166	2
	79.7	24.3	104	30	66	1
	37.9	92.8	130.7	45	118	2
	53.7	10.98	64.68	50	159	1
	145.3	34.38	179.68	33	78	1
	87.3	15.71	103.01	64	204	2
	90.3	60.1	150.4	91	205	2
	76	22.02	98.02	39	111	2
	98.6	20.3	118.9	23	145	1
	58.6	23.36	81.96	87	118	4
	443.5	55.5	499	51	291	2
	56	19.33	75.33	31	91	1
	133.75	61.8	195.55	82	201	2
	91.19	29	120.19	42	151	1
